# Outcomes of Care by Geriatricians and Non-geriatricians in an Academic Hospital

**DOI:** 10.3389/fmed.2022.908100

**Published:** 2022-06-06

**Authors:** Reshma Aziz Merchant, Vanda Wen Teng Ho, Matthew Zhixuan Chen, Beatrix Ling Ling Wong, Zhiying Lim, Yiong Huak Chan, Natalie Ling, Shu Ee Ng, Amelia Santosa, Diarmuid Murphy, Anantharaman Vathsala

**Affiliations:** ^1^Division of Geriatric Medicine, Department of Medicine, National University Hospital, Singapore, Singapore; ^2^Department of Medicine, Yong Loo Lin School of Medicine, National University of Singapore, Singapore, Singapore; ^3^Biostatistics Unit, Yong Loo Lin School of Medicine, National University of Singapore, Singapore, Singapore; ^4^Division of Rheumatology, Department of Medicine, National University Hospital, Singapore, Singapore; ^5^Value Driven Outcomes Office, National University Health System, Singapore, Singapore; ^6^Division of Nephrology, Department of Medicine, National University Hospital, Singapore, Singapore

**Keywords:** geriatric medicine, cost, length of stay, readmission, mortality

## Abstract

**Introduction:**

While hospitalist and internist inpatient care models dominate the landscape in many countries, geriatricians and internists are at the frontlines managing hospitalized older adults in countries such as Singapore and the United Kingdom. The primary aim of this study was to determine outcomes for older patients cared for by geriatricians compared with non-geriatrician-led care teams.

**Materials and Methods:**

A retrospective cohort study of 1,486 Internal Medicine patients aged ≥75 years admitted between April and September 2021 was conducted. They were either under geriatrician or non-geriatrician (internists or specialty physicians) care. Data on demographics, primary diagnosis, comorbidities, mortality, readmission rate, Hospital Frailty Risk Score (HFRS), Age-adjusted Charlson Comorbidity Index, Length of Stay (LOS), and cost of hospital stay were obtained from the hospital database and analyzed.

**Results:**

The mean age of patients was 84.0 ± 6.3 years, 860 (57.9%) females, 1,183 (79.6%) of Chinese ethnicity, and 902 (60.7%) under the care of geriatricians. Patients under geriatrician were significantly older and had a higher prevalence of frailty, dementia, and stroke, whereas patients under non-geriatrician had a higher prevalence of diabetes and hypertension. Delirium as the primary diagnosis was significantly higher among patients under geriatrician care. Geriatrician-led care model was associated with shorter LOS, lower cost, similar inpatient mortality, and 30-day readmission rates. LOS and cost were lower for patients under geriatrician care regardless of frailty status but significant only for low and intermediate frailty groups. Geriatrician-led care was associated with significantly lower extended hospital stay (OR 0.73; 95% CI 0.56–0.95) and extended cost (OR 0.69; 95% CI 0.54–0.95).

**Conclusion:**

Geriatrician-led care model showed shorter LOS, lower cost, and was associated with lower odds of extended LOS and cost.

## Introduction

Worldwide, especially in countries with fast-aging populations, a significant proportion of acute hospital beds are occupied by older adults. Multimorbidity, polypharmacy, dementia, and frailty are highly prevalent in hospitalized older adults (1). Older adults are heterogenous, present atypically, and are at higher risk for hazards of hospitalization. There is a shortage of geriatricians across many countries and acute care for older adults is typically provided by hospitalists, internists, family physicians, and/or geriatricians. In the United States, hospitalist-led care dominates whereas in the United Kingdom, acute care is provided by internists or geriatricians.

Healthcare systems were designed traditionally to provide care for patients with an acute illness which require rapid diagnosis without much focus on older adults with varied functional, nutritional, and psychosocial needs. Cognitive impairment and/or frailty are highly prevalent in older inpatients where some studies have reported in excess of 30% (2–4). Frailty is a multidimensional syndrome, a state of decreased physiological reserve predisposing older adult to adverse outcomes such as delirium, falls, pressure ulcers, functional decline, drug–drug and drug–disease interactions (5). In older adults, age and acute illness are not the only determinants of prognosis but also their existing comorbidities, cardiorespiratory status, and functional and cognitive limitations. Frailty is an important predictor of healthcare outcomes and requires a targeted, and effective strategy for the delivery of care across the frailty continuum (5). Comprehensive geriatric assessment (CGA) is the recommended standard of care for prioritizing multidimensional care for older adult with frailty. CGA is proven effective, with numbers needed to treat 20 for mortality or institutionalization in hospitalized older adults (6). CGA has been shown to reduce inappropriate prescribing, polypharmacy and overall medication cost (7). However, performing this assessment for all the hospitalized older patients can be resource-intensive. In this regard, healthcare systems worldwide are studying the use of frailty score derived from electronic health records such as electronic frailty index (FI) or Hospital Frailty Risk Score (HFRS) to guide resource allocation (8–10).

Vulnerable older adults are often underrepresented in clinical trials. This lack of evidence complicates the management of older adults, where less is often more and more is less (11). Older adults typically present with geriatric syndromes such as immobility, instability (falls), incontinence, and impaired intellect (dementia/delirium) initially coined by Bernard Isaacs (12). Multiple geriatric syndromes can co-exist in the same patient, where almost half of the patient older than 80 years have four or more geriatric syndromes (13). In addition to age, prevalence of geriatric syndromes is also highly prevalent in patients with dementia where 90.3% of patients with dementia with Lewy bodies and 54.9% with Alzheimer’s disease have three or more geriatric syndromes (14). Training the future physicians on applying evidence and prioritizing management in older adults with varied trajectories of frailty and cognition is crucial to reduce unnecessary harm from over-treatment (15, 16). Managing acute illness in older adults is both an art and skill which requires structured training programs. Unlike geriatric fellowship training in the United States which lasts a year, training for geriatricians in Singapore lasts 3 years encompassing core clinical geriatrics of 2 years and rotations to hospice care (inpatient and home care), complex rehabilitation facilities, subacute care facilities, nursing homes, and attachments to geriatric subspecialties (continence, cognition, ortho-geriatrics, geriatric oncology), home care, and community programs on healthy aging.

Geriatric approach to older acute medical patients with greater emphasis on “what matters to me,” rehabilitation and discharge planning has been associated with shorter length of stay (LOS) and possibly reduced need for institutionalization, though these were seen only in small trials (17). The aim of this study was to determine if patients under geriatricians had a shorter LOS and incurred lower cost compared with non-geriatricians (internists and sub-specialists) within an academic hospital.

## Methodology

We conducted a retrospective study using data obtained from the hospital medical records database at National University Hospital (NUH), a tertiary hospital in the western part of Singapore. All the older patients (aged ≥75 years) were included if they were admitted under Internal Medicine in fourteen different wards between April and September 2021. Geriatricians were the primary attending physicians for older patients admitted into four wards, and care for older patients admitted to the remaining wards was provided by either internists or ward-based sub-specialists. Patients admitted to Acute Medical Unit and isolation wards were excluded. Informed consent requirement was waived because of the retrospective nature of the study and deidentified data were obtained from hospital database. The study was reviewed and approved by the National Healthcare Group Domain Specific Review Board.

### Demographic Data

In addition to patients’ age, gender, ethnicity, underlying comorbidities, and primary diagnosis, we obtained data on Age-adjusted Charlson comorbidity Index and HFRS. Data on LOS, cost, in-hospital mortality, 30-day readmission, and 30-day mortality was obtained from the database. Cost was further sub-categorized into laboratory, radiology, and medication-related costs. Primary diagnosis was defined by International Classification of Disease 10 (ICD-10) codes (18). LOS, total cost, and breakdown by laboratory, medications, and radiology was calculated for nine common diagnoses of delirium, pneumonia, fragility fracture, urinary tract infection (UTI), infections, stroke, intracranial hemorrhage, congestive cardiac failure, and myocardial infarction.

Hospital Frailty Risk Score was initially described by Gilbert et al. and utilizes ICD-10 codes to generate a frailty risk score (10). HFRS has been validated in multiple patient populations and is predictive of LOS, inpatient mortality, adverse events, and cost (8, 19). Age-adjusted Charlson Comorbidity Index is a constitute of weighted index of age, number, and seriousness of comorbid disease, and originally validated to predict mortality (20). Extended LOS and cost were defined as those with more than 75th percentile for LOS and cost, respectively.

### Statistical Analysis

IBM SPSS Version 27.0 was used for analysis with statistical significance set at two-sided 5%. Descriptive analyses were presented as frequencies with percentages for categorical variables and mean with SD or median and interquartile range for continuous variables. Significance testing by Pearson χ^2^ test for categorical and Mann–Whitney *U*-tests for continuous variables were conducted. Multivariate logistic regression model for clinical binary outcomes were adjusted for age, Charlson Comorbidity Index and Frailty. Odds ratios with 95% CIs were presented.

## Results

### Patients’ Characteristics

Between April and September 2021, 1,486 patients were admitted under Internal Medicine where 902 (60.7%) were under the care of geriatricians and 584 (39.3%) under non-geriatricians (Table 1). The mean age of patients was 84.0 ± 6.3 years, 860 (57.9%) women and 1,183 (79.6%) of Chinese ethnicity. Delirium (18.3%) and pneumonia (15.5%) were the top two primary diagnoses, followed by UTI (10.7%), infection (7.2%), fragility fractures defined by vertebral, humeral, hip and compression fractures (3.4%), congestive cardiac failure (3.4%), intracranial bleed (1.6%), myocardial infarction (1.4%), and stroke (0.7%). Most frequent co-existing conditions include hypertension (45.0%), diabetes (35.8%), and chronic kidney disease (32.4%). For the frailty indices, 45.5% were classified as low on HFRS, 41.3% intermediate, and 13.3% high HFRS. The median age-adjusted Charlson Comorbidity Index was 6 (IQR 3.0). The overall in-hospital mortality was 7%, 30-day mortality 6.8%, and 30-day readmission 10.2%. The mean LOS was 7.17 ± 6.8 days and mean cost SGD $5,932.7 ± 4,770.0.

**TABLE 1:** Demographics and outcomes.

	All	Geriatrician-led care	Non-geriatrician led care	*P*-value
	1486 (100%)	902 (60.7%)	584 (39.3%)	
Demographics				
**Gender**				
Male	626 (42.1)	378 (41.9)	248 (42.5)	0.436
Female	860 (57.9)	524 (58.1)	336 (57.5)	
Age (years)	84.0 ± 6.3	85.3 ± 6.2	84.1 ± 6.4	**<0.001**
**Ethnicity**				0.131
Chinese	1183 (79.6)	743 (82.4)	440 (75.3)	**0.016**
Malay	125 (8.4)	61 (6.8)	64 (11.0%)	
Indian	112 (7.5)	65 (7.2)	56 (9.6)	
Others	54 (3.6)	31 (3.4)	23 (3.9)	
**Principal diagnosis**				
Delirium	272 (18.3)	240 (26.6)	32 (5.5)	**<0.001**
Pneumonia	230 (15.5)	130 (14.4)	100 (17.1)	0.091
Urinary tract infection	159 (10.7)	72 (8.0)	87 (14.9)	**<0.001**
Infection	106 (7.2)	55 (6.1)	51 (8.8)	0.063
Fracture (vertebral, shoulder and hip)	50 (3.4)	27 (3.0)	23 (3.9%)	0.377
Stroke	11 (0.7)	9 (1.0)	2 (0.3)	0.218
Intracranial bleed	24 (1.6)	13 (1.4)	11 (1.9)	0.532
Congestive cardiac failure	51 (3.4)	24 (2.7)	27 (4.6)	0.057
Myocardial infarction	21 (1.4)	15 (1.7)	6 (1.0)	0.373
**Co-existing conditions**				
Diabetes	532 (35.8)	303 (33.6)	229 (39.2)	**0.016**
Hypertension	669 (45.0)	387 (42.9)	282 (48.3)	**0.024**
Dementia	228 (15.3)	155 (17.2)	73 (12.5)	**0.008**
CKD	482 (32.4)	288 (31.9)	194 (33.2)	0.322
Cancer	16 (1.1)	9 (1.0)	7 (1.2)	0.450
Stroke	41 (2.8)	32 (3.5)	9 (1.5)	**0.014**
Intracranial bleed	14 (0.9)	7 (0.8)	7 (1.2)	0.422
Ischemic heart disease	45 (3.0)	23 (2.5)	22 (3.8)	0.119
Osteoporosis	141 (9.5)	88 (9.8)	53 (9.1)	0.366
Compression fracture	39 (2.6)	26 (2.9)	13 (2.2)	0.508
Falls as primary or secondary diagnosis	249 (16.9%)	160 (17.7%)	89 (15.2%)	0.227
Hospital frailty risk score [median (IQR)]		6.1 (10.6)	5.3 (8.0)	0.050
Low	676 (45.5)	390 (43.2)	286 (49.0)	**<0.001**
Intermediate	613 (41.3)	366 (40.6)	247 (42.3)	
High	197 (13.3)	146 (16.2)	51 (8.7)	
Age-adjusted charlson comorbidity index [median (IQR)]	6.0 (3.0)	6.0 (3.0)	6.0 (4.0)	0.880
Physiotherapy review				**0.022**
No	273 (18.4)	152 (16.9)	121 (20.7)	
0–1 days	598 (40.2)	387 (42.9)	211 (36.1)	
≥2 days	615 (41.4%)	363 (40.2)	252 (43.2)	

**Outcomes**				

In-hospital Mortality	104 (7.0)	63 (7.0)	41 (7.0)	1.000
30-day Mortality	101 (6.8)	71 (8.5)	30 (5.1)	**0.045**
30-day Readmission	152 (10.2)	98 (11.7)	54 (9.9%)	0.462
**Length of stay**				
Mean	7.2 ± 6.8	6.7 ± 6.3	7.9 ± 7.4	**0.003**
Median (IQR)	5.0 (6.0)	5.0 (5)	6.0 (6)	**<0.001**
**Cost (total)**				
Mean	$5933 ± $4770	$5617 ± $4313	$6420 ± $5368	**0.002**
Median (IQR)	$4546 ± $4935	$4429 ± 4116	$4697 ± 5038	**0.006**
**Cost**				
Nasogastric tube (mean ± SD)*	$33 ± $165	$20 ± 116	$53 ± 219	**<0.001**
Urinary Catheter (mean ± SD)	$5 ± $21	$4 ± $18	$7 ± $25	**0.008**

*NGT tube inserted in 5.4% of geriatrician-led team vs. 9.2% in non-geriatrician–led team, mean ± SD.

*$: SGD$. Bolded: p < 0.05.*

Patients under geriatricians were significantly older (85.3 ± 6.2 vs. 84.1 ± 6.4 years) compared with non-geriatricians. Delirium as primary diagnosis was significantly higher among older patients cared for by geriatricians (26.6%) compared with non-geriatricians (5.5%). On the other hand, UTI as primary diagnosis was significantly higher among patients cared for by non-geriatricians (14.9%) compared with geriatricians (8.0%).

Almost 1 in 5 older patients under geriatricians had underlying dementia compared with 1 in 8 under non-geriatricians. Patients under geriatricians had significantly higher co-existing chronic condition of stroke, whereas those under non-geriatricians had higher prevalence of hypertension and diabetes. Among patients admitted under geriatricians, 16.2% had high HFRS compared with 8.7% under non-geriatricians with no difference in the median age-adjusted Charlson Comorbidity Index. For time to physiotherapy review, 54.4% under non-geriatricians were seen in 2 or more days compared with 48.4% of older patients under geriatricians.

### Outcomes

**FIGURE 1:**
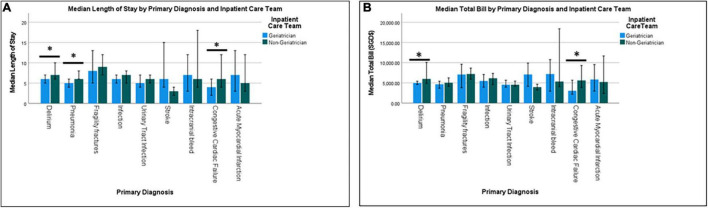
Median length of stay **(A)** and median total bill **(B)** by primary diagnosis and inpatient care team.

There was no significant difference for in-hospital mortalities between the groups, but 30-day mortality was significantly higher for patients discharged from geriatrician-led care teams (8.5 vs. 5.1%, respectively). Mean LOS was significantly lower among patients under geriatricians (6.7 ± 6.3 days) compared with non-geriatricians (7.9 ± 7.4 days). Patients with primary diagnosis of delirium, pneumonia, and congestive cardiac failure had significantly shorter LOS when cared for by geriatricians (Figure 1).

**FIGURE 2:**
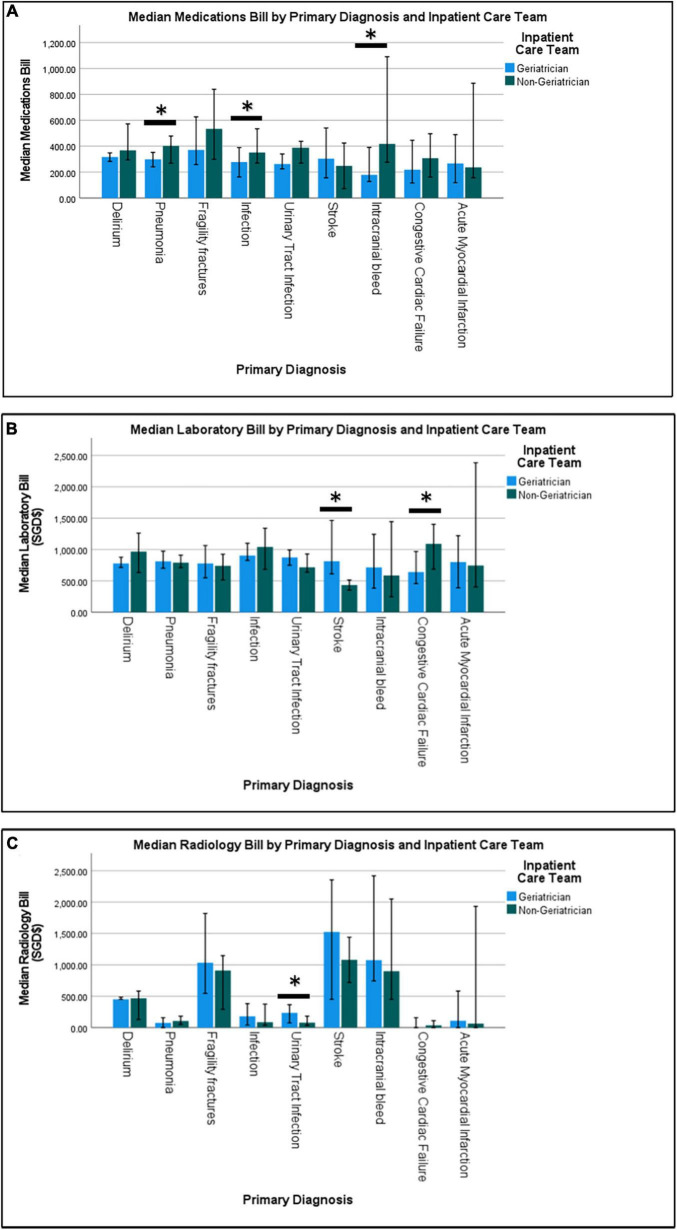
Median medications bill **(A)**, median laboratory bill **(B)** and median radiology bill **(C)** by primary diagnosis and inpatient care team.

**FIGURE 3:**
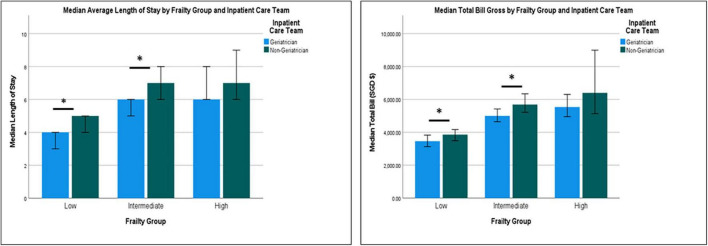
Median length of stay and total bill by frailty group and inpatient care team.

Similarly, patient cared for by geriatricians had lower total cost (SGD $5,617 ± 4,313 vs. $6,420 ± 5,368), lower nasogastric tube (NGT) (SGD $120 ± 116 vs. $53 ± 219) and catheter-related costs (SGD $4 ± 18 vs. $7 ± 25). Total median cost for patients under geriatricians was significantly lower for delirium (SGD $4,985, IQR 4,412 vs. $5,995, IQR 5,762) and congestive cardiac failure ($3,066, IQR 3,991 vs. SGD $5,618, IQR 6,192). Patients under geriatricians had significantly higher laboratory cost for stroke (SGD $813, IQR 737 vs. $434, IQR 162) but lower for congestive cardiac failure (SGD $641, IQR 662 vs. $1,090, IQR 784) (Figure 2). While radiological cost for UTI was significantly higher for patients under geriatricians (SGD $236, IQR 758 vs. $80, IQR 378), medications cost was significantly lower for pneumonia (SGD $299, IQR $324 vs. $402, IQR $387), infection (SGD $278, IQR $373 vs. $352, IQR $562) and intracranial bleed (SGD $179, IQR $218 vs. $418, IQR $662). LOS was lower for patients under geriatricians across the frailty status but significant only for low- and intermediate-frailty groups (Figure 3).

In the adjusted multiple regression model (Table 2), geriatrician-led care was associated with significantly lower extended hospital stay (OR 0.73; 95% CI 0.56–0.95) and extended cost (OR 0.69; 95% CI 0.54–0.95).

**TABLE 2:** Logistic regression on association of geriatrician-led vs. non-geriatrician–led inpatient team on readmission within 30 days, in-hospital mortality, cost, and length of stay (non-geriatrician–led as reference group).

Predictors	Unadjusted OR (95% CI) *p*-value	Adjusted OR (95% CI) *p*-value
In-hospital mortality	0.994 (0.661–1.495) 0.979	0.953 (0.632–1.438) 0.819
30-day mortality	**1.578 (1.016–2.450) 0.042**	1.490 (0.956–2.322) 0.078
Readmission within 30 days	1.196 (0.843–1.697) 0.315	1.202 (0.846–1.708) 0.304
eLOS	0.828 (0.645–1.064) 0.140	**0.731 (0.564–0.947) 0.018**
eCOST	**0.774 (0.610–0.982) 0.035**	**0.690 (0.539–0.882) 0.003**

*eLOS: Extended hospital length of stay (>75th percentile study population).*

*eCOST: Extended cost (>75th percentile study population).*

*Adjusted for frailty, age-adjusted charlson comorbidity index. Bolded: p < 0.05.*

## Discussion

Healthcare transformation from volume to value requires measurements such as cost and outcomes. Geriatrician-led care for older adults aged ≥75 years was significantly associated with lower cost and shorter LOS with no significant increase in inpatient mortality or 30-day readmission. More than half of the patients under geriatrician care had intermediate or high HFRS and slightly more than a quarter had delirium as primary diagnosis. Delirium as primary diagnosis was only prevalent in 5.5% of patients under non-geriatricians. Various small-scale studies under trial conditions have shown geriatric inpatient service to be associated with shorter LOS, improved functional status, lower prevalence of polypharmacy, and lower laboratory and/or medication-related cost (17, 21). Our study is one of the largest to show the benefits of geriatric care for older hospitalized patients.

Patients under the care of geriatricians with pneumonia and/or delirium had better outcomes such as shorter LOS and lower cost, especially for total bill for delirium and medication bill for pneumonia. Delirium is known to be present in 20–40% of older patients in medical wards (2, 3). Delirium is often poorly recognized and under-diagnosed leading to prolonged LOS, functional decline, and discharge to nursing home (2, 3). LOS for patients with delirium in our study was 6.1 days, possibly due to the efficient subacute care community rehabilitation facilities where patients receive rehabilitation in a more cost-effective setting (22). Dementia, frailty, and advanced age are all significant risk factors for delirium, and presence of delirium warrants early multidisciplinary approach to the management of such patients. While insignificant, the prevalence of pneumonia was lower in patients under geriatrician care despite higher numbers of patients with high HFRS and dementia. The lower NGT and urinary catheter costs with fewer patients under geriatricians having NGT and urinary catheter insertion further supports the role of CGA and multidisciplinary care in reducing tethers which are known to precipitate delirium, promote immobility with subsequent functional decline. Feeding tubes are not recommended in persons with dementia, having been associated with agitation and greater use of physical and chemical restraints (23).

Despite higher HFRS being associated with increased 30-day mortality, longer LOS and 30-day readmission (8, 10), this was not seen in patients under geriatrician care, where despite higher prevalence of patients with high HFRS, LOS was significantly lower with no significant increase in 30-day readmission. In total, thirty-day mortality was increased for patients under geriatrician-led care team which could be attributed to the higher mean age and HFRS for patients under geriatrician care.

At the population level, prevalence of frailty is 6.2% and pre-frailty 37.0% where polypharmacy is prevalent in almost half of the frail older adults (24). The negative consequences of metabolic syndrome in older adults is mediated by frailty, and polypharmacy is present in 71.4% of these frail older adults (25). Depending on the tools used, prevalence of inpatient frailty is between 25.0 and 65.2% (26, 27). Older adults with frailty are known to have higher comorbidity burden and polypharmacy and often excluded from clinical trials. The Systolic Blood Pressure Intervention Trial (SPRINT), while it included participants with frailty with a median FI of 0.18 (less fit), those with underlying diabetes and stroke were excluded (28). A 1% increase in FI was associated with increased odds of self-reported falls, injurious falls, and all-cause hospitalization (29). Training of medical students and specialists in the area of frailty assessment with individualized management is crucial as following guidelines without tailoring to life expectancy, functional status, and patient preference may be harmful for the patient. Tinetti et al. in emphasized on “shift from disease-centered to patient goals–directed care” for patient value-based care where domain examples included function and mobility, symptoms, life prolongation, wellbeing/quality of life and occupational or social roles (30).

In addition to being a good clinician who provides cost-effective and evidence-based care and educator, geriatricians are known for their diverse roles including administrators in acute care and community settings, researchers and advocates for quality care for older adults across the healthcare settings which is crucial to maintain the standards of care across institution (31). Geriatricians are also known to be the most satisfied physicians (32) but there still continues to be a shortage of geriatricians and many healthcare systems have implemented initiatives to improve care of older adults through collaborative care models such as ortho-geriatric units, geriatric trauma units, acute care for elders (ACEs) units, CGA units, and delirium units (31, 33–35). While it is recommended that older adults get admitted to specific units where staffs are trained to conduct CGA, and manage the vulnerable older adults, with increasing number of hospitalized older patients, Age-Friendly Health Systems focusing on “What Matters,” “Medication,” “Mentation,” and “Mobility” (36) or the Geriatric Resource Nurse Care model may be a better concept (37). Given the shortage of geriatricians, and many older adults continue to be admitted under sub-specialties such as cardiology, oncology, trauma, and various surgical disciplines, one of the most important roles of a geriatrician is to educate other physicians, recommending assessment tools such as Rapid Geriatric Assessment that screens for frailty, sarcopenia, and anorexia of aging and cognition or electronic FI and putting in place a system for timely referrals of patient who are at risk of hazards of hospitalization and/or disability (9, 38). NUH has implemented the Nurses Improving Care of Healthsystem Elders (NICHEs) model (39) and introduced Eldercare Bundle hospital wide. Eldercare Bundle is a structured assessment and multidisciplinary management process, and involves daily review of physical, nutrition, cognitive, psychosocial function by ward nurses with implementation of protocol to improve or maintain continence, nutrition, mobility, skincare, mood, cognition, and sleep. In addition, for patients who are confused or agitated are nursed in SILVER Unit (Specialized Innovative LongeVity and Elderly Recovery) with zero restraint policy. Despite the various initiatives for patients under non-geriatricians, geriatrician-led care was associated with better outcomes.

While our studies have many strengths such as robust database and inclusion of all patients admitted under Internal Medicine, there are limitations which warrant mention. Due to retrospective nature of the study, we did not have information on the functional, nutrition, admission and discharge medications, and psychosocial aspects including care burden and caregiver stress for the patients. No causal inferences can be made from retrospective data. Second, the accuracy of data retrieved is subject to accuracy of coding. Third, non-geriatricians within the institution do receive support from ward-based geriatric resource nurses which could have an impact on overall outcome. At last, HFRS does not capture the fluctuations caused by acute illness and hence may underestimate the actual functional consequence.

Morley in commented that despite geriatricians function as super-specialists, in some countries they are under-appreciated and get little respect and suggested for geriatricians “to stop being shrinking violets and to loudly proclaim” (31). This article has shown that geriatricians are definitely a valuable asset in an academic institution, more so in the times of COVID-19 pandemic and thereafter (40). Geriatrician-led care was associated with lower cost and shorter LOS especially for delirium, pneumonia, congestive cardiac failure, and infection overall despite the increased prevalence of patients with high HFRS. While the easiest solution would be to have all older adults cared for by geriatricians, the model is clearly unsustainable with the current numbers of geriatricians and increasing older acute inpatients. With greater visibility on cost and outcomes for individual, primary diagnosis will allow us to focus on improving value for the conditions where cost remains high.

## Conclusion

With populations aging worldwide and increase in demand for age-friendly healthcare systems, geriatrician presence in improving healthcare outcomes in older adults is crucial. Geriatrician-led care was associated with shorter LOS, lower cost with no significant difference in in-hospital mortality and 30-day readmissions. Geriatrician-led care was cost effective across the frailty status, most significant for the intermediate and lower frailty groups.

## Data Availability Statement

The raw data supporting the conclusions of this article will be made available by the authors, without undue reservation.

## Ethics Statement

The studies involving human participants were reviewed and approved by the National Healthcare Group Domain Specific Review Board. Written informed consent for participation was not required for this study in accordance with the national legislation and the institutional requirements.

## Author Contributions

RM conducted the data acquisition. RM, VH, and YC conducted the data analysis and interpretation. All authors contributed to study concept, design, and preparation of manuscript.

## Conflict of Interest

The authors declare that the research was conducted in the absence of any commercial or financial relationships that could be construed as a potential conflict of interest.

## Publisher’s Note

All claims expressed in this article are solely those of the authors and do not necessarily represent those of their affiliated organizations, or those of the publisher, the editors and the reviewers. Any product that may be evaluated in this article, or claim that may be made by its manufacturer, is not guaranteed or endorsed by the publisher.
